# CRISPR/Cas9-mediated targeted gene correction in amyotrophic lateral sclerosis patient iPSCs

**DOI:** 10.1007/s13238-017-0397-3

**Published:** 2017-04-11

**Authors:** Lixia Wang, Fei Yi, Lina Fu, Jiping Yang, Si Wang, Zhaoxia Wang, Keiichiro Suzuki, Liang Sun, Xiuling Xu, Yang Yu, Jie Qiao, Juan Carlos Izpisua Belmonte, Ze Yang, Yun Yuan, Jing Qu, Guang-Hui Liu

**Affiliations:** 1 National Laboratory of Biomacromolecules, CAS Center for Excellence in Biomacromolecules, Institute of Biophysics, Chinese Academy of Sciences, Beijing, 100101 China; 2 State Key Laboratory of Stem Cell and Reproductive Biology, Institute of Zoology, Chinese Academy of Sciences, Beijing, 100101 China; 3 University of Chinese Academy of Sciences, Beijing, 100049 China; 4 Department of Molecular and Cellular Physiology, Stanford University School of Medicine, Stanford, CA 94305 USA; 5 Department of Neurology, Peking University First Hospital, Beijing, 100034 China; 6 Gene Expression Laboratory, Salk Institute for Biological Studies, 10010 North Torrey Pines Road, La Jolla, CA 92037 USA; 7 Universidad Católica San Antonio de Murcia (UCAM), Campus de los Jerónimos, N 135 Guadalupe, 30107 Murcia, Spain; 8 Department of Gynecology and Obstetrics, Peking University Third Hospital, Beijing, 100191 China; 9 Beijing Hospital of the Ministry of Health, Beijing, 100730 China; 10 Key Laboratory of Regenerative Medicine of Ministry of Education, Institute of Aging and Regenerative Medicine, Jinan University, Guangzhou, 510632 China; 11 Beijing Institute for Brain Disorders, Capital Medical University, Beijing, 100069 China

**Keywords:** ALS, CRISPR/Cas9, gene correction, iPSC disease modeling

## Abstract

Amyotrophic lateral sclerosis (ALS) is a complex neurodegenerative disease with cellular and molecular mechanisms yet to be fully described. Mutations in a number of genes including *SOD1* and *FUS* are associated with familial ALS. Here we report the generation of induced pluripotent stem cells (iPSCs) from fibroblasts of familial ALS patients bearing *SOD1* ^+/*A272C*^ and *FUS* ^+/*G1566A*^ mutations, respectively. We further generated gene corrected ALS iPSCs using CRISPR/Cas9 system. Genome-wide RNA sequencing (RNA-seq) analysis of motor neurons derived from *SOD1* ^+/*A272C*^ and corrected iPSCs revealed 899 aberrant transcripts. Our work may shed light on discovery of early biomarkers and pathways dysregulated in ALS, as well as provide a basis for novel therapeutic strategies to treat ALS.

**Electronic supplementary material:**

The online version of this article (doi:10.1007/s13238-017-0397-3) contains supplementary material, which is available to authorized users.

## Introduction

Amyotrophic lateral sclerosis (ALS) are a group of progressive but fatal neurodegenerative diseases due to the selective loss of functional motor neurons in the brain and spinal cord. On average, most ALS patients die within 5 years once symptom appears. Excitotoxic/Inflammatory/Oxidative insults, misfolded proteins and aggregates, aberrant RNA processing, unstable genome, and mitochondria dysfunction have all been implicated in ALS (Pasinelli and Brown, 2006; Kiskinis et al., 2014). Despite extensive studies over the past 40–50 years, the etiology of ALS is still far from clear. ALS is generally classified into two categories: familial ALS (FALS) and sporadic ALS (SALS). Two forms are clinically similar. FALS accounts for 10% of total ALS cases and is mostly dominant inheritance (Robberecht and Philips, 2013). To date, many genes have been implicated in FALS, such as *SOD1*, *FUS*, *TDP43*, *C9ORF72*, *VAPB*, etc. Gene *SOD1* encoding superoxide dismutase 1 (SOD1) is the first identified gene linked to FALS and accounts for about 20% of FALS cases (Rosen et al., 1993). Mutant SOD1 was found as aberrant misfolded aggregates in FALS (Bruijn et al., 1998; Bosco et al., 2010). *FUS* was initially identified in cancer and encodes a RNA/DNA binding protein (Crozat et al., 1993; Baechtold et al., 1999). A number of mutations in *FUS* gene were identified in ALS cases, like A1564G, C1574T, G1566A, etc. (Drepper et al., 2011; Lai et al., 2011; Lattante et al., 2013). Aggregates containing mutant FUS protein have been found in motor neurons from ALS patients by postmortem analysis (Vance et al., 2009).

Although great progresses have been achieved in studying animal models of ALS, the clinical relevance of many observations in animals is uncertain. For instance, most of the rodent ALS models are transgenic animals carrying extremely high levels of the specific proteins when compared with human ALS pathological tissues (Julien and Kriz, 2006; Turner and Talbot, 2008; Huang et al., 2011; Sharma et al., 2016). Likewise, many early events of neuronal dysfunction and retraction have been well documented in animal models; however, correlated timings of those events in human ALS pathology especially of earlier ones are unclear (Fischer et al., 2004; Casci and Pandey, 2015). Moreover, the anti-excitotoxic agent Riluzole is by far the only compound discovered through ALS animal models, but it only demonstrated moderate therapeutic effect in clinical trials (Zhu et al., 2015). The genetic and developmental differences between human and model species urge more understanding in human system. Recently, vast progresses in using human induced pluripotent stem cells (iPSCs) and gene engineering tools to model human genetic diseases have brought promising prospects (Liu et al., 2011a; Liu et al., 2011b; Egawa et al., 2012; Liu et al., 2012a; Liu et al., 2012b; Yang et al., 2013; Liu et al., 2014; Suzuki et al., 2014; Zhang et al., 2015; Kubben et al., 2016). A few groups reported the establishment of novel ALS disease models by combining iPSCs and gene engineering tool (Chen et al., 2014; Kiskinis et al., 2014; Wainger et al., 2014; Lenzi et al., 2015; Higelin et al., 2016; Ichiyanagi et al., 2016). Other ALS iPSCs disease models were also reported however lacked isogenic gene corrected controls; thus the underlying mechanism may be obscured due to variable genetic backgrounds (Egawa et al., 2012; Li et al., 2015; Liu et al., 2015). More importantly, none of these studies focused on the early molecular events in ALS pathogenesis.

Here, we report the generation of iPSCs from ALS patients carrying heterozygous mutations of *SOD1* ^+/*A272C*^ and *FUS* ^+/*G1566A*^, respectively. We also achieved targeted correction of the mutant genes via combing CRISPR/Cas9 and single-stranded oligodeoxynucleotide (ssODN) without leaving any genomic footprint. Using a modified motor neuron differentiation method, we subsequently generated ISL1^+^ motor neurons from isogenic iPSC lines and performed genome-wide RNA sequencing (RNA-seq) analysis. To the end, we identified a group of aberrant transcripts which may be involved in *SOD1* ^*+*/*A272C*^-mediated pathogenesis of ALS. Thus, our study provides an isogenic platform to study ALS disease mechanisms and cues to develop novel therapies.

## Results

### Establishment of *SOD1*^+/*A272C*^ and *FUS*^+/*G1566A*^ iPSCs

**Figure 1:**
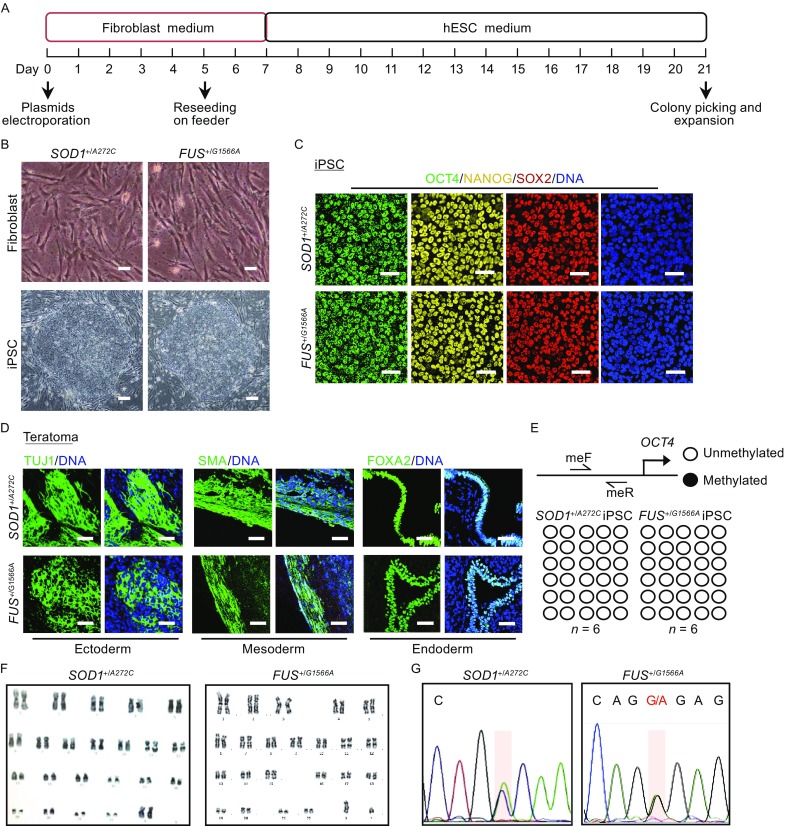
Establishment of *SOD1* ^+/*A272C*^ iPSCs and *FUS* ^+/*G1566A*^ iPSCs. (A) Schematic procedure of generating iPSCs from ALS patient fibroblasts. (B) Phase-contrast images of ALS patient fibroblasts (top panels) and iPSCs (bottom panels). Scale bars = 25 µm (top bottom) and 100 μm (bottom panels). (C) Immunofluorescent staining of pluripotency markers, OCT4, NANOG, and SOX2. Nuclei were stained with Hoechst 33342 (blue). Scale bars = 50 μm. (D) Immunofluorescent staining of TUJ1 (ectoderm), SMA (mesoderm), and FOXA2 (endoderm) in teratomas derived from ALS iPSCs *in vivo*. Nuclei were stained with Hoechst 33342 (blue). Scale bars = 50 μm. (E) DNA methylation analysis of the *OCT4* promoter in ALS iPSCs. A pair of primers used is shown as arrows. Open and closed circles indicate unmethylated and methylated CpG dinucleotides respectively, as indicated. (F) Karyotyping analysis of ALS patient iPSCs. (G) Confirmation of the heterozygous mutation of *SOD1* ^+/*A272C*^ and *FUS* ^+/*G1566A*^ in ALS iPSCs by DNA sequencing

We obtained primary fibroblasts with a heterozygous missense mutation (A272C) in *SOD1* gene and a heterozygous nonsense mutation (G1566A) in *FUS* gene from two ALS patients, respectively. We then generated *SOD1* ^+/*A272C*^ specific iPSCs (*SOD1* ^+/*A272C*^ iPSCs) and *FUS* ^+/*G1566A*^ specific iPSCs (*FUS* ^+/*G1566A*^ iPSCs) according to a modified reprogramming protocol (Takahashi et al., 2007; Okita et al., 2011; Liu et al., 2014; Ding et al., 2015; Fu et al., 2016) (Fig. 1A). About 20 human embryonic stem cell (hESC)-like colonies were generated from 5 × 10^5^ starting fibroblasts. We did not observed discernible difference in reprogramming efficiency between the two lines of patients-derived fibroblasts (data not shown). The generated iPSCs displayed typical pluripotent stem cell-like morphology, expressed pluripotency markers, such as OCT4, NANOG and SOX2, formed teratomas consisting of three germ-layers *in vivo*, maintained unmethylated CpG islands in the promoter of *OCT4*, and demonstrated normal karyotypes (Fig. 1B–F). DNA sequencing results demonstrated the presence of *SOD1* ^+/*A272C*^ or *FUS* ^+/*G1566A*^ mutations in the two lines of ALS iPSCs, respectively (Fig. 1G). These data collectively supported the successful generation of *SOD1* ^+/*A272C*^ and *FUS* ^+/*G1566A*^ iPSCs from ALS patients’ fibroblasts.

### Targeted gene correction of *FUS* and *SOD1* mutations with CRISPR/Cas9 system

**Figure 2:**
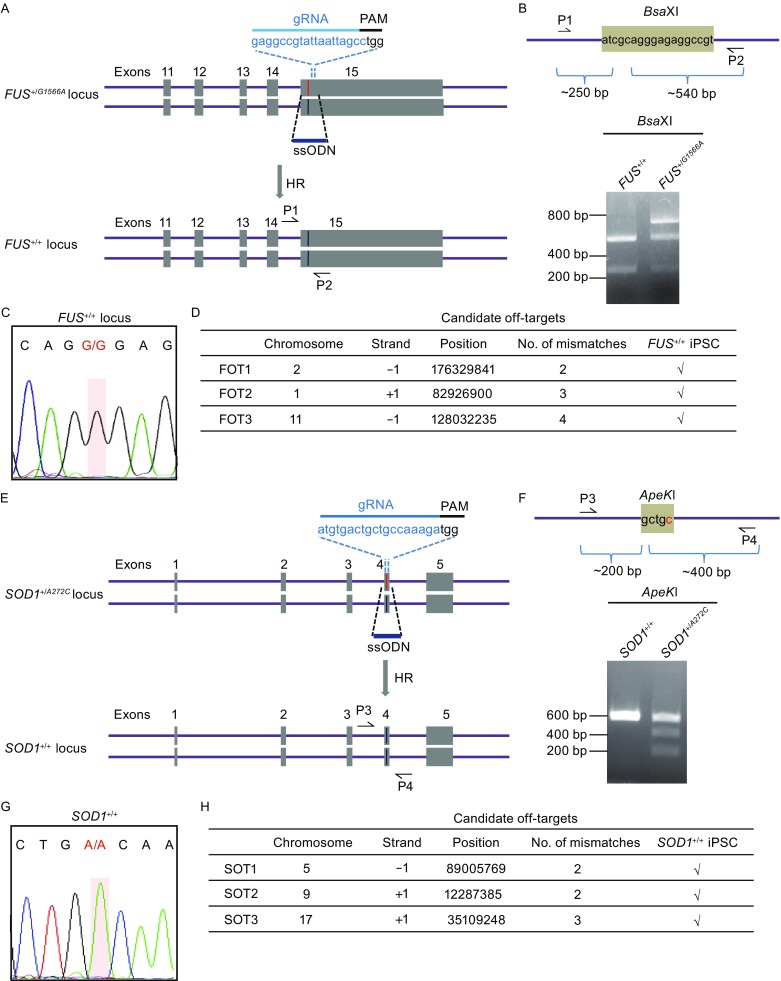

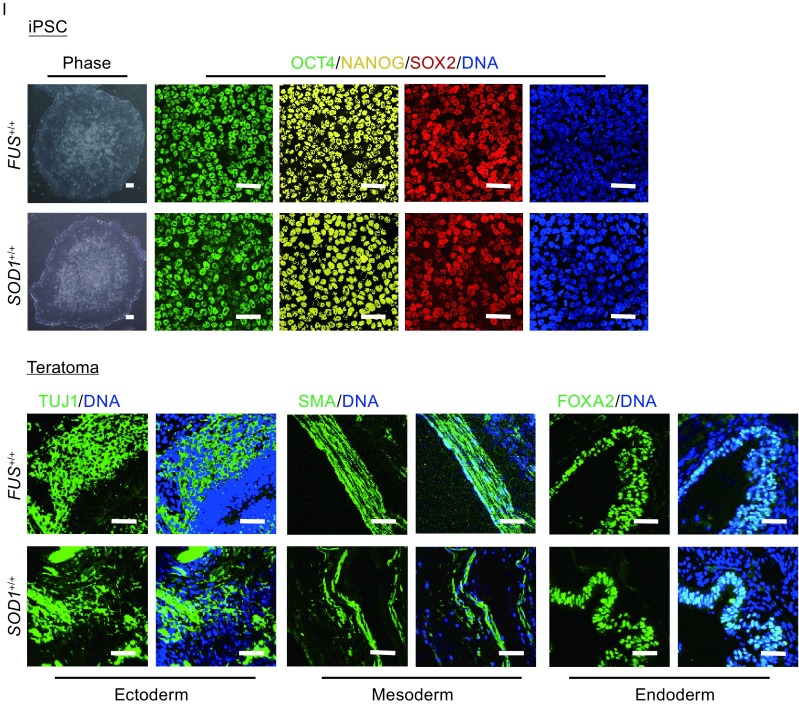
Targeted gene correction of *FUS* and *SOD1* mutations. (A) Strategy of correcting *FUS* ^+/G1566A^ mutation. The sequence of gRNA is shown with the PAM sequence. Red line represents the mutant allele, and blue line represents the wildtype allele. HR, homologous recombination. ssODN, single-stranded oligodeoxynucleotide. (B) *Bsa*XI restriction digestion of PCR product before and after gene correction. The red letter highlights the corrected base. The mutation G>A eliminates *Bsa*XI restriction site that is present in the corrected line. Primers used are shown as arrows in Fig. 2A (P1, P2). (C) DNA sequencing demonstrates the correction of *FUS* ^+/*G1566A*^ mutation. The red shadow highlights the corrected base. (D) DNA sequencing of the top 3 potential off-target sites. The off-target sites were predicated at http://crispr.genome-engineering.org/. Sequencing of PCR product shows no off-targets found. FOT, potential off-target site at gRNA targeted *FUS* gene. +1, plus strand. −1, minus strand. √, no off targets. (E) Strategy of correcting *SOD1* ^+/A272C^ mutation. The sequence of gRNA is shown with the PAM sequence. Red line represents the mutant allele, and blue line represents the wildtype allele. HR, homologous recombination. ssODN, single-stranded oligodeoxynucleotide. (F) *Ape*KI restriction digestion of PCR product before and after gene correction. The red letter highlights the mutant base. The mutation A>C creates *Ape*KI restriction site that is absent in the corrected line. Primers used are shown as arrows in Fig. 2E (P3, P4). (G) DNA sequencing demonstrates the correction of *SOD1* ^+/*A272C*^ mutation. The red shadow highlights the corrected base. (H) DNA sequencing of the top 3 potential off-target sites. The off-target sites were predicated at http://crispr.genome-engineering.org/. Sequencing of PCR product shows no off-targets found. SOT, potential off-target site at gRNA targeted *SOD1* gene. +1, plus strand. −1, minus strand. √, no off targets. (I) Immunofluorescent images of pluripotency markers, OCT4, NANOG, and SOX2 incorrected clone *in vitro* and three-layer markers, TUJ1 (ectoderm), SMA (mesoderm), and FOXA2 (endoderm) in teratomas derived from corrected iPSCs *in vivo*. Nuclei were stained with Hoechst 33342 (blue). Scale bars = 50 μm

In order to circumvent clonal variations of iPSC lines and better elucidate the pathogenic mechanism of the ALS mutations, we sought to generate isogenic wild type iPSC lines by targeted gene correction. Using CRISPR/Cas9 system and a donor plasmid containing wild type coding sequence and homology arms (HA) as the repair template (Fig. S1A), we obtained the gene corrected iPSC clone (*FUS* ^+/+^-HA iPSCs) from *FUS* ^+/*G1566A*^ iPSCs. After homologous recombination and the subsequent removal of neo-resistance cassette, a flippase recognition target site (FRT footprint) was left in *FUS* intron. Site-specific gene correction was confirmed by genomic DNA PCR and sequencing (Fig. S1B and S1C). Additionally, *FUS* ^+/+^-HA iPSCs demonstrated normal pluripotency (Fig. S1D). Recent studies have shown that linear templates with short (60–70 bases) homology arms, like single-stranded oligodeoxynucleotide (ssODN), could also be used in gene correction instead of plasmid-based construct. This strategy bypasses the selection cassette insertion and leaves no genetic footprint (Corti et al., 2012; Ding et al., 2013). We next tested this strategy by electroporating an expression vector encoding both mCherry and guide RNA, a plasmid for Cas9-2A-GFP, and the ssODN template all together into patient iPSCs. After fluorescence-activated cell sorting (FACS) for GFP (Cas9) and mCherry (guide RNA) double positive cells, gene corrected clones (*FUS* ^+/+^ iPSCs) were successfully obtained (Fig. 2A). The *Bsa*XI restriction fragment length polymorphism (RFLP) was specific to the corrected allele and DNA sequencing also confirmed that *FUS* ^+/*G1566A*^ was correctly targeted without any footprint (Figs. 2B, 2C, and S2A.). The gene targeting efficiency at *FUS* gene was about 1% (Fig. S2A). In view of the potential clinical application of gene editing technique (Suzuki et al., 2014; Veres et al., 2014), we further investigated the specificity of gene editing in the system. We carefully examined the top 3 potential off-target sites and found no unwanted off-target effect (Figs. 2D and S3A). Comparing both gene correction platforms, we found no obvious difference regarding targeting efficiency (data not shown). Accordingly, we chose to correct *SOD1* ^+/*A272C*^ via CRISPR/Cas9 system using ssODN as repair template (Fig. 2E). Correction of *SOD1* ^+/*A272C*^ was confirmed by genomic DNA sequencing, as well as elimination of *Ape*K1 RFLP (Figs. 2F, 2G, and S2B). The gene targeting efficiency at *SOD1* gene was about 20% (Fig. S2B). We did not find off-target cleavages in *SOD1* ^+/+^ iPSCs by checking the predicted top 3 off-target sites as well (Figs. 2H, S3B, and S4). Additionally, both corrected iPSCs expressed pluripotency markers including OCT4, NANOG, and SOX2 *in vitro,* and formed teratomas composed of three germ layers *in vivo* (Fig. 2I). Taken together, we successfully generated gene corrected ALS iPSCs displaying normal pluripotency.

### Directed motor neuron differentiation from isogenic pair of iPSC lines

**Figure 3:**
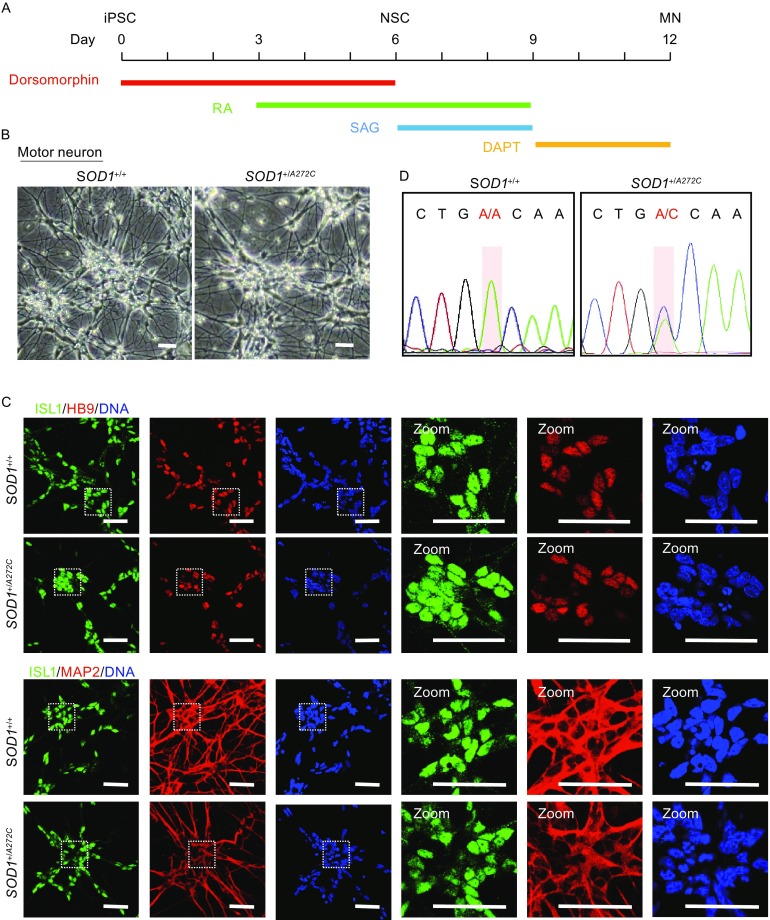
Directed motor neuron differentiation from isogenic pair of iPSC lines. (A) Schematic overview of motor neuron differentiation from iPSCs. Compounds are added at different time points as indicated. NSC, neural stem cell. MN, motor neuron. (B) Phase contrast images of motor neurons derived from *SOD1* ^+/*A272C*^ iPSCs and its isogenic control iPSCs at day 12. Scale bars = 75 μm. (C) Immunofluorescent images of motor neuron markers (ISL1, HB9) and other neural marker (MAP2) at day 12. Nuclei were stained with Hoechst 33342 (blue). Scale bars = 50 μm. (D) DNA Sequencing confirming *SOD1* genotype in motor neurons derived from *SOD1* ^+/*A272C*^ iPSCs and its isogenic control iPSCs at day 12

The cardinal symptom of ALS patients is muscle atrophy due to the dysfunction of motor neurons (Al-Chalabi and Hardiman, 2013). To investigate the biological effect of ALS-causing genetic mutations, we generated motor neurons from *SOD1* ^+/*A272C*^ iPSCs and corresponding isogenic controls using a modified protocol with high differentiation efficiency (Qu et al., 2014; Maury et al., 2015) (Fig. 3A). During the differentiation, four key compounds were sequentially added, including dorsomorphin for neural induction, retinoic acid (RA), and smo agonist (SAG) to activate neural patterning, and DAPT (γ-secretase inhibitor) to accelerate motor neurons maturation. Upon day 12, most cells showed neuronal morphology (Fig. 3B). Nearly all of differentiated cells were MAP2^+^, about 70%–80% cells were ISL1^+^, and up to 70% of the cells were HB9^+^ (Figs. 3C and S5A). Mutation and genetic correction were further confirmed in differentiated motor neurons by sequencing (Fig. 3D). It has been reported that wildtype and SOD1 mutant iPSC lines may have comparable neuronal differentiation capacity (Kiskinis et al., 2014). Similarly, we did not observe significant difference in the efficiency of motor neuron generated from ALS iPSCs and their wildtype isogenic controls (Figs. 3C and S5A).

### RNA-seq revealed *SOD1*^+/*A272C*^-affected pathways in human motor neurons

**Figure 4:**
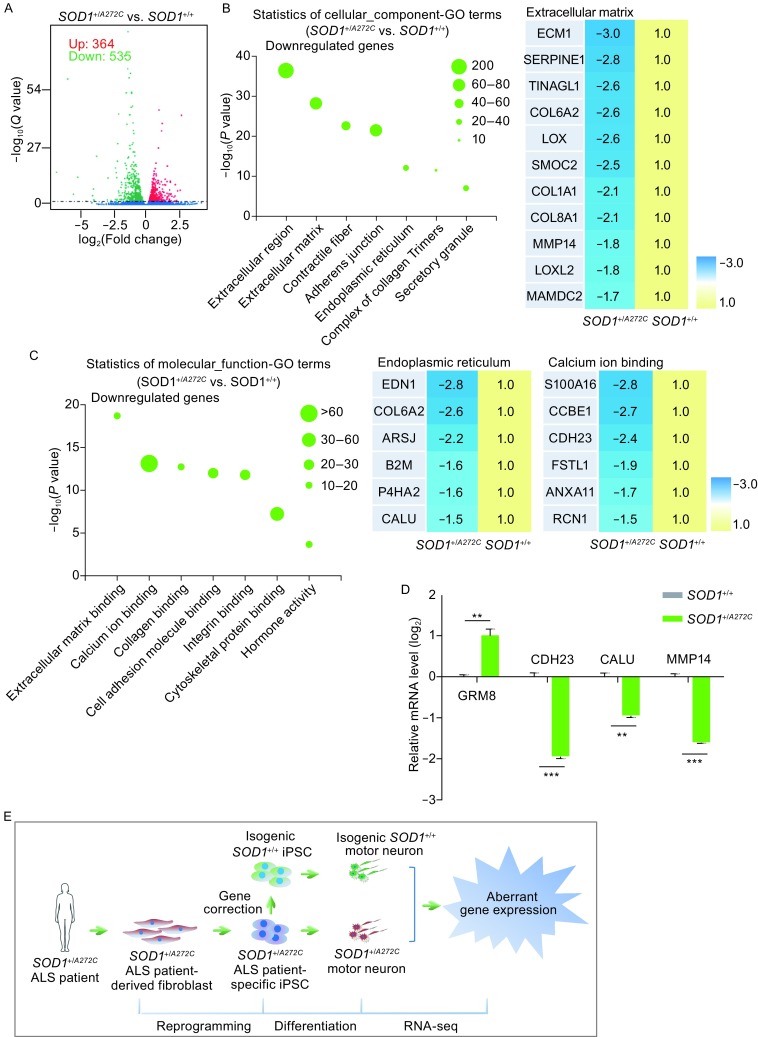
RNA-seq revealed *SOD1* ^+/*A272C*^-affected early pathways in human motor neurons. (A) Volcano plot analysis showing significantly altered genes (*q* value < 0.05) between *SOD1* ^+/*A272C*^ and its isogenic control motor neurons. 535 downregulated genes (green) and 364 upregulated genes (red) were found in *SOD1* ^+/*A272C*^ motor neurons compared with its isogenic control motor neurons. (B and C) GO terms based cellular_component and molecular_function enrichment analysis of the significantly downregulated gene sets in *SOD1* ^+/*A272C*^ motor neurons. Heatmap of dysregulated genes between *SOD1* ^+/*A272*C^ motor neurons and its isogenic control motor neurons. Number of altered genes in each GO term is indicated by size of the bubble. (D) RT-qPCR analysis of dysregulated genes in *SOD1* ^+/*A272C*^ motor neurons and its isogenic control motor neurons. Values were normalized against *GAPDH*. Data were presented as mean ± SEM, *n* = 4, *P < 0.05, **P < 0.01, ***P < 0.001. (E) A proposed strategy generating ALS disease model using iPSC, gene editing, and cell differentiation approaches. Fibroblasts are obtained from ALS patient bearing *SOD1* ^+/*A272C*^ mutation and reprogrammed to iPSCs. Isogenic control is created via gene editing. RNA-seq is performed in motor neurons to uncover *SOD1* ^+/*A272C*^-affected early events underlying ALS pathogenesis. RNA-seq analysis shows *SOD1* ^+/*A272C*^ mutation induces aberrant gene expression

In order to better understand the molecular mechanisms of ALS-causing mutations especially those molecular events at early stage of ALS pathogenesis, we performed RNA-seq analysis to compare global gene expression in motor neurons carrying *SOD1* mutation with isogenic wild type controls. Filtering by setting *q*-values less than 0.05, we identified 364 upregulated genes and 535 downregulated genes in *SOD1* ^+/*A272C*^ motor neurons (Figs. 4A, S5B, S5C, and Table S1). Gene ontology analysis revealed that upregulated transcripts of *SOD1* ^+/*A272C*^ motor neurons were associated with regulation of nervous system activity and signal transduction, etc. (Fig. S5D, S5E, and Table S2). Downregulated transcripts were associated with the terms of extracellular matrix, calcium homeostasis, and endoplasmic reticulum (ER) homeostasis, etc. (Figs. 4B, 4C, S5E, and Table S2). Key gene expression changes were verified by RT-qPCR (Fig. 4D). These gene expression changes may be implicated in early molecular events accounting for ALS pathogenesis.

## Discussion

Basic research in ALS animal models has provided valuable insights into the mechanism of the disease. However, knowledge we learned from animal models were often poorly transferable into clinic. Amongst all variable factors, large genetic variations among different species and strategy of overexpressing mutant genes in animal models may account for major gaps between animal models and clinical cases of ALS. Generating human iPSCs bearing disease mutation together with an efficient neuronal differentiation strategy may provide an unprecedented tool to study cellular and molecular mechanisms of ALS in human neurons. Here, we generated two human iPSC lines from ALS patient-specific fibroblasts bearing heterozygous disease-causing mutations (*FUS* ^+/*G1566A*^ and *SOD1* ^+/*A272C*^), and generated their respective isogenic disease-free iPSCs by CRISPR/Cas9 mediated gene correction.

Previous studies have reported genetic correction of *SOD1* ^+/*A272C*^ mutation and *SOD1* ^+/*C14T*^ mutation in ALS patients derived iPSCs. Notably, these research teams employed TALEN- and ZFN-mediated gene editing together with correction vectors containing drug resistance cassette, resulting in FRT footprint in the engineered genome (Chen et al., 2014; Kiskinis et al., 2014). In our study, we generated isogenic gene-corrected iPSCs using CRISPR/Cas9 system with ssODN as repair template for correcting *SOD1* ^+/*A272C*^ and *FUS* ^+/*G1566A*^ into wildtypes. Using ssODN but not plasmids with resistance cassette in our strategy simplified the gene correction procedure, and avoided introducing any exogenous sequence into the genome. This strategy offers a great potential for generating other human genetic disease models as well.

Motor neuron differentiation protocols were reported from embryoid body based strategy to single cell based differentiation (Boulting et al., 2011; Qu et al., 2014; Du et al., 2015). There are apparent limitations regarding the complicacy of differentiation procedure and the purity of neurons generated. By optimizing the combination of chemical molecules and simplifying extracellular matrix, we developed a robust and easy-handle method to produce human ISL1^+^ motor neurons at around 70% purity. As we learned from animal models, biological changes of ALS started early in life however the manifestation of the disease may become clinically apparent much later. For example, the dysfunction of neuromuscular junction (NMJ) and the loss of synapse and axons occur before onset of ALS symptom. The initial alterations are not well studied due to the lack of a precisely-controlled disease model (Conforti et al., 2007; Kanning et al., 2010). Taking advantage of iPSC and directed cell differentiation *in vitro*, it is now feasible to collect different phases of motor neurons bearing ALS-related mutation to comprehensively investigate pathogenesis of ALS including early events. Notably, differences in gene expression profiles of ALS motor neurons without obvious degeneration were carefully explored and directly compared to their gene-corrected controls in our study. Therefore, our ALS iPSC disease model has a great value and advantage in studying early events of ALS progression.

*SOD1* ^+/*A272C*^ encodes for an aspartic acid to alanine mutation in SOD1 protein, and it is a prevalent mutation in Europe ALS patients. In contrast, *FUS* ^+/*G1566A*^ bears an arginine to arginine nonsense alteration. Although this mutation was reported in clinical cases (Drepper et al., 2011; Lai et al., 2011), the underlying pathogenic mechanism was unknown. Our work demonstrated for the first time that both *SOD1* ^+/*A272C*^ and *FUS* ^+/*G1566A*^ mutations are able to be corrected by CRISPR/Cas9-mediated gene editing technique in ALS patient specific iPSCs. We also developed an isogenic iPSC disease model for studying the pathogenic effect of *SOD1* ^+/*A272C*^ on early stage of motor neuron pathology. Comparing ALS motor neurons with their disease-free controls, we found that several pathways may be essentially implicated in SOD1-associated early ALS pathogenesis. In *SOD1* ^+/*A272C*^ motor neurons, we identified transcriptional changes in a subset of genes involved in signal transduction, organization of extracellular matrix, cellular homeostasis maintenance, and neurogenesis, etc. Dysfunctional cell-cell signaling has been reported as one of the causes contributing to death of motor neurons. For example, toxic factors secreted from astrocytes progressively damage motor neurons in ALS (Nagai et al., 2007). Previous study showed that GRM3 and GRM5, which are glutamate metabotropic receptors belonging to G protein-coupled receptors (GPCR) family, are highly expressed in human ALS pathological specimens. This observation indicates that cell communication might play critical regulatory roles in underlying ALS pathogenesis (Aronica et al., 2001). Our data showed that GRM8, another member of GPCR, was upregulated in *SOD1* ^+/*A272C*^ motor neurons as well. Notably, the role of glutamate-mediated excitotoxicity in ALS pathogenesis has been well studied, and the only FDA-approved ALS-treated drug Riluzole aims at decreasing the level of glutamate (Zhu et al., 2014).

Loss of calcium homeostasis is another factor which may contribute to motor neuron death in ALS cases (Bruijn et al., 2004). Many calcium-binding proteins were downregulated in ALS patient autopsy specimens (Alexianu et al., 1994). In this study, we identified that calcium-binding protein CDH23, a calcium dependent cell-cell adhesion glycoproteins, was reduced in *SOD1* ^+/*A272C*^ motor neurons. In addition, calumenin (CALU), another calcium-binding protein that safeguards ER homeostasis (Lee et al., 2013), decreased in *SOD1* ^+/*A272C*^ motor neurons. Both dysfunction of calcium signaling and increased ER stress have been linked to ALS-associated neurodegeneration at early stages (Lee et al., 2016; Woehlbier et al., 2016) .

Extracellular matrix has been shown to influence neuronal degeneration (Soleman et al., 2013; De Luca and Papa, 2016). However the role of matrix metalloproteinases in neuronal disorder diseases has not been well established (Cirillo et al., 2016). We identified that matrix metalloproteinase 14 (MMP14) decreased in *SOD1* ^+/*A272C*^ motor neurons. Consistently, a previous study identified that MMP14 significantly decreased in SOD1^G93A^ transgenic mice and SALS patients (Kudo et al., 2010). Thus, the role of extracellular matrix in ALS pathogenesis may represent an interesting topic for future study.

Enhanced neurogenesis in ALS transgenic mice model has been recently reported (Chi et al., 2007; Lee et al., 2011). While neural stem cells (NSCs) isolated from pre-symptomatic ALS transgenic mice proliferate and differentiate well, NSCs from late stage ALS mice lose their functional activities (Lee et al., 2011). Our RNA-seq data also supported that ALS was associated with a tendency of increased neurogenesis at gene expression level, despite that no obvious phenotypic defect was observed.

In summary, our study successfully established valuable isogenic ALS iPSC disease models using CRISPR/Cas9-mediated gene editing. More importantly, we identified potential molecular hallmarks associated with early pathological events of motor neuron with *SOD1* ^+/*A272C*^ ALS mutation. The experimental system established in our report may hold a potential for further mechanistic study, drug screening, and inspire autologous therapy against ALS in the future (Fig. 4E).

## Materials and methods

### Cell culture

Human ALS patient fibroblasts (ND29149, heterozygous for *SOD1* ^+/*A272C*^; ND29563, heterozygous for *FUS* ^+/*G1566A*^) were obtained from Coriell Institute (http://www.coriell.org). All fibroblasts were maintained in high glucose DMEM (Invitrogen) supplemented with 15% fetal bovine serum (FBS, Hyclone), 1% glutamax (Invitrogen), 1% non-essential amino acids (Invitrogen), 1% penicillin/streptomycin (Invitrogen). iPSCs were maintained on a layer of mitomycin C-inactivated mouse embryonic fibroblast (MEF) with cDF12 medium or on Matrigel (BD Biosciences) with mTeSR1 medium (Stem Cell Technologies) as previously described (Liu et al., 2014; Ding et al., 2015; Fu et al., 2016).

### iPSC generation

ALS fibroblasts were reprogrammed using the episomal vectors as described (Okita et al., 2011; Liu et al., 2014; Fu et al., 2016).

### Plasmid construction

Guide RNA (gRNA) was designed in http://crispr.mit.edu. gRNAs were cloned into pCAGmCherry-gRNA (Suzuki et al., 2016). pCAG-1BPNLS-Cas9-1BPNLS-2AGFP was used as Cas9 and GFP expression plasmid (Suzuki et al., 2016). For the experiments using CRISPR/Cas9 system together with a plasmid donor including neo, a gRNA_cloning vector (Mali et al., 2013) was employed, and the homology arms were cloned into Neo-pCR2.1 vector (donor plasmid) as previously described (Duan et al., 2015).

Primers for gRNA are listed as follows: *FUS*-gRNA-F, 5′-GAAGGAAAATTAACTCAGG-3′; *FUS*-gRNA-R, 5′-CTGAGTTAATTTTCCTTCC-3′; *FUS*-gRNA-mCherry-F, 5′-GAGGCCGTATTAATTAGCC-3′; *FUS*-gRNA-mCherry-R, 5′-GGCTAATTAATACGGCCTCC-3′; *SOD1*-gRNA-mCherry-F, 5′-ATGTGACTGCTGCCAAAGA-3′; *SOD1*-gRNA-mCherry-R, 5′-CTTTGGCAGCAGTCACATC-3′. Primers for homology arms are listed as follows: *FUS*-LA-F, 5′-ATAGGGCCCTGGTACTGAGGTATGTGCGTGTTTTCCAAAGAA-3′; *FUS*-LA-R, 5′-CCCTCGAGTTAATTTTCCTTCCCTCTCCACTACTGGTTACAAC-3′; *FUS*-RA-F, 5′-CGGGATCCCTCAGGGGGAGTGAATCTGTAGACCCAC-3′; *FUS*-RA-R, 5′-CCCAAGCTTCTCAAGCCCTCTGAGTACAGGCAGGATG-3′. The ssODNs used to repair mutant allele are listed as follows: *FUS*-ssODN, 5′-ACCTGGGGAGCCAGGCTAATTAATACGGCCTCTCCCTGCGATCCTGTCTGTGCTCACCCCTGC-3′; *SOD1*-ssODN, 5′-TCTTCAATAGACACATCGGCCACACCATCTTTGTCAGCAGTCACATTGCCCAAGTCTCCAACAT-3′.

### Targeted gene correction in ALS iPSCs via CRISPR/Cas9 system

Correction of *FUS* ^+/*G1566A*^ mutation via homologous recombination was carried out as previously described with some modifications (Duan et al., 2015; Pan et al., 2016).

In brief, *FUS* ^+/*G1566A*^ iPSCs cultured on Matrigel using mTeSR1 medium were pre-treated with ROCK inhibitor Y-27632 (Sigma) overnight and dissociated with TrypLE (Invitrogen). For electroporation, 5 × 10^6^ cells were collected. Cells were resuspended in 100 μL Opti-Mem (Gibco) containing 7 μg Cas9, 7 μg gRNA-FUS, and 7 μg donor plasmids. After electroporation, cells were plated on mitomycin C-inactivated DR4-MEF feeder. Two days after electroporation, G418 (50 μg/mL, Gibco) were added to enrich gene-targeted cells. After additional two weeks, G418-resistant clones were picked and verified by genomic PCR and DNA sequencing. The correct clones were expanded and used for further experiments. Primers used for identifying correct clones are listed as followed: P5, 5′-AATGATACCAGTTGCTTGATGGATACTAGGTGCTT-3′; P6, 5′-ACCTTTCTGCTCTTGGGTTAATGTTACGCTCT-3′. Neomycin-resistance cassette was removed from gene-targeted iPSCs as previously described (Duan et al., 2015; Pan et al., 2016).

Gene correction with single-stranded oligodeoxynucleotide (ssODN) as repair template was carried out as previously described with some modifications (Peters et al., 2013). Briefly, 5 × 10^6^ iPSCs were resuspended in 100 μL Opti-Mem (Gibco), containing 8 μg Cas9-2A-GFP, 4 μg gRNA-mCherry, and 8 μg ssODN. After electroporation, cells were plated on Matrigel-coated plate and cultured with mTeSR1 medium. 48 hours after electroporation, mCherry^+^/GFP^+^ cells were collected by FACS and reseeded on MEF feeder. Two weeks later, clones were picked and screened by RFLP and DNA sequencing. Primers used are listed as follows: P1-*FUS*-S-F, 5′-GAGAAAGTGGTTTCATTTTGAGGGCTAGGTGGA-3′; P2-*FUS*-S-R, 5′-TTGTTTGAGCCTCACCATTAAAAGGGCCAAAAG-3′; P3-*SOD1*-S-F, 5′-CCCATCTTTCTTCCCAGAGCATTAGTGTGTAGACG-3′; P4-*SOD1*-S-R, 5′-ACAAAATGTTCTGTTTAACAAGTGAGAAACCCAATCCT-3′.

### Motor neuron differentiation

Motor neuron differentiation was performed as previously described with some modifications (Liu et al., 2014; Maury et al., 2015). Briefly, iPSCs were passaged onto MEF feeder and then motor neuron differentiation was initialized. Medium 1 containing 50% advanced DMEM/F12 (Invitrogen), 50% neurobasal (Invitrogen), 1× N2 (Invitrogen), 1× B27 (Invitrogen), 1% GlutaMAX (Invitrogen), 1% NEAA (Invitrogen), 1% penicillin/streptomycin (Invitrogen), 4 μmol/L CHIR99021 (Cellagentech), 3 μmol/L SB431542 (Cellagentech), 1 μmol/L dorsomorphin (Sigma), and 0.1 μmol/L compound E (EMD Chemicals), 0.2% heparin (Sigma) was used for the first 3 days. Then, RA (100 nmol/L) was added to the medium for another 3 days (medium 2). On day 6, dorsomorphin was then removed, and SAG (500 nmol/L) was added (medium 3). At day 8, cells were dissociated to single cells with Accumax (Millipore) and reseeding on Matrigel-coated plates. DAPT and laminin were added on day 9 to accelerate MN differentiation.

### Teratoma assay

5 × 10^6^ iPSCs were injected subcutaneously into NOD-SCID mice (male, 6–8 weeks old). After 8–12 weeks of injection, teratomas were dissected and analyzed by immunostaining (Liu et al., 2014; Fu et al., 2016; Ren et al., 2017). All murine experiments were conducted in compliance with animal protocols approved by the Chinese Academy of Science Institutional Animal Care and Use Committee.

### Immunofluorescence

Cells were washed once using PBS, fixed with 4% paraformaldehyde for 30 min at room temperature, permeabilized with 0.4% Triton X-100 in PBS for 20 min and were blocked in donkey serum (10% in PBS) for 60 min. cells were incubated with primary antibodies overnight in 4°C. Then, the cells were stained with secondary antibodies and Hoechst 33342 at room temperature. The antibodies used in immunofluorescence assay are as follows: anti-NANOG (Abcam, 21624, 1:200), anti-OCT3/4 (Santa Cruz, 5279, 1:100), anti-SOX2 (Santa Cruz,17320, 1:100), anti-TUJ1 (Sigma, T2220, 1:500), anti-FOXA2 (CST, 8186, 1:200), anti-SMA (Sigma, A5228, 1:200), anti-ISL1 (Abcam, ab20670 1:250), anti-HB9 (DSHB, 81.5C10, 1:50), anti-MAP2 (Sigma, 4403, 1:500).

### Bisulfite sequencing analysis

Bisulfite sequencing of the *OCT4* promoter was carried out as previously described (Liu et al., 2011a; Duan et al., 2015). Primers used are listed as follows: meF-*OCT4*, 5′-ATTTGTTTTTTGGGTAGTTAAAGGT-3′; meR-*OCT4*, 5′-CCAACTATCTTCATCTTAATAACATCC-3′.

### Off-target analysis

Off-target analysis was performed following a previous study with modifications (Liang et al., 2015). The off-target sites were predicated in http://crispr.genome-engineering.org/. The top 3 off-target sites were analyzed by DNA sequencing. Primers used are listed as follows: *FUS*-OT1-F, 5′-AGCTTCTGCCAAGATCTGGTTTCTTCGTC-3′; *FUS*-OT1-R, 5′-TTTCAAAGACACACACCACCCTGACCAT-3′; *FUS*-OT2-F, 5′-ACCTGCCATCATAGTCTAGTATCGTTCTCT-3′; *FUS*-OT2-R, 5′-AACAATCGACCCACTCCCATCATGACC-3′; *FUS*-OT3-F, 5′-ACATTTCTGGCTCAGCTTCAATCATGGT-3′; *FUS*-OT3-R, 5′-ATTCCTTGGCTTGAAGATTATAGGTGAC-3′; *SOD1*-OT1-F, 5′-TTCAATGGCATGTAGGGAAGGACCAAAGTTGAA-3′; *SOD1*-OT1-R, 5′-ATAGCTCTATAAATGCCAGCTGTTGAAGGCAGG-3′; *SOD1*-OT2-F, 5′-ATATTCAGATGGCCTGAATGTCCAGATGCTGTG-3′; *SOD1*-OT2-R, 5′-CTCACAAGTAGGGTGATAACAGCTGCCATACTG-3′; *SOD1*-OT3-F, 5′-CCCTTCTCCAGCCTTACTCTTTCCATATAGCC-3′; *SOD1*-OT3-R, 5′-TCTGAGCCTGCTTTAAGTCCCAGACACGTT-3′.

### RT-qPCR

Total RNA was extracted from cells by TRIzol Reagent (Invitrogen). Around 2 μg total RNA was employed for cDNA synthesis with reverse transcription Master Mix (Promega). RT-qPCR was performed using iTaq Universal SYBR Green Supermix (Bio-Rad) on a CFX384 Real-Time PCR system (Bio-Rad). All data were normalized by *GAPDH* transcript and calculated using the ∆∆Cq method. Primers used for RT-qPCR are listed in Table S3.

### RNA-seq library construction

Motor neurons were collected for constructing sequencing library. Total RNA was isolated with TRIzol Reagent (Invitrogen). After qualified by Fragment Analyzer (Advanced Analytical), 3 μg RNA was used to construct sequencing library NEBNext® Ultra™ RNA Library Prep Kit for Illumina® (NEB, USA) following manufacturer’s recommendations (Li et al., 2016).

### RNA-seq data analysis

Clean data (clean reads) were obtained from raw data (raw reads) by removing reads containing adapter, reads containing ploy-N and low quality reads (>50% of reads with Q phred ≤ 20) from raw data. Then reads were mapped to the Homo sapiens hg19 genome (UCSC) by Tophat2 (Trapnell et al., 2009). Differential expression analysis was performed using the DESeq R package (1.18.0) (Anders and Huber, 2010).

Gene Ontology (GO) enrichment analysis of differentially expressed genes was implemented by the GOseq R package, in which gene length bias was corrected. GO terms with corrected *P* value less than 0.05 were considered significantly enriched by differential expressed genes (Anders and Huber, 2010).

### Statistical analysis

Results were presented as mean ± SEM. Two tailed Student’s *t*-test was conducted using Graph-Pad Prism Software. *P* values < 0.05 were considered statistically significant (*).

### Accession number

All of the RNA-seq data have been deposited in GEO under the accession number GSE95089.

## Electronic supplementary material

Below is the link to the electronic supplementary material.

Supplementary material 1 (PDF 589 kb)

Supplementary material 2 (XLSX 40 kb)

Supplementary material 3 (XLSX 40 kb)

Supplementary material 4 (XLS 24 kb)
