# Supplementary material for: CRISPR/Cas9-mediated targeted gene correction in amyotrophic lateral sclerosis patient iPSCs
Source: Protein Cell. 2017 Apr 11;8(5):365–78. doi: 10.1007/s13238-017-0397-3 (PMC5413600; doi:10.1007/s13238-017-0397-3)
Supplement: Supplementary file 1 — Supplementary material 1 (PDF 589 kb) [file 13238_2017_397_MOESM1_ESM.pdf]

Figure S1.

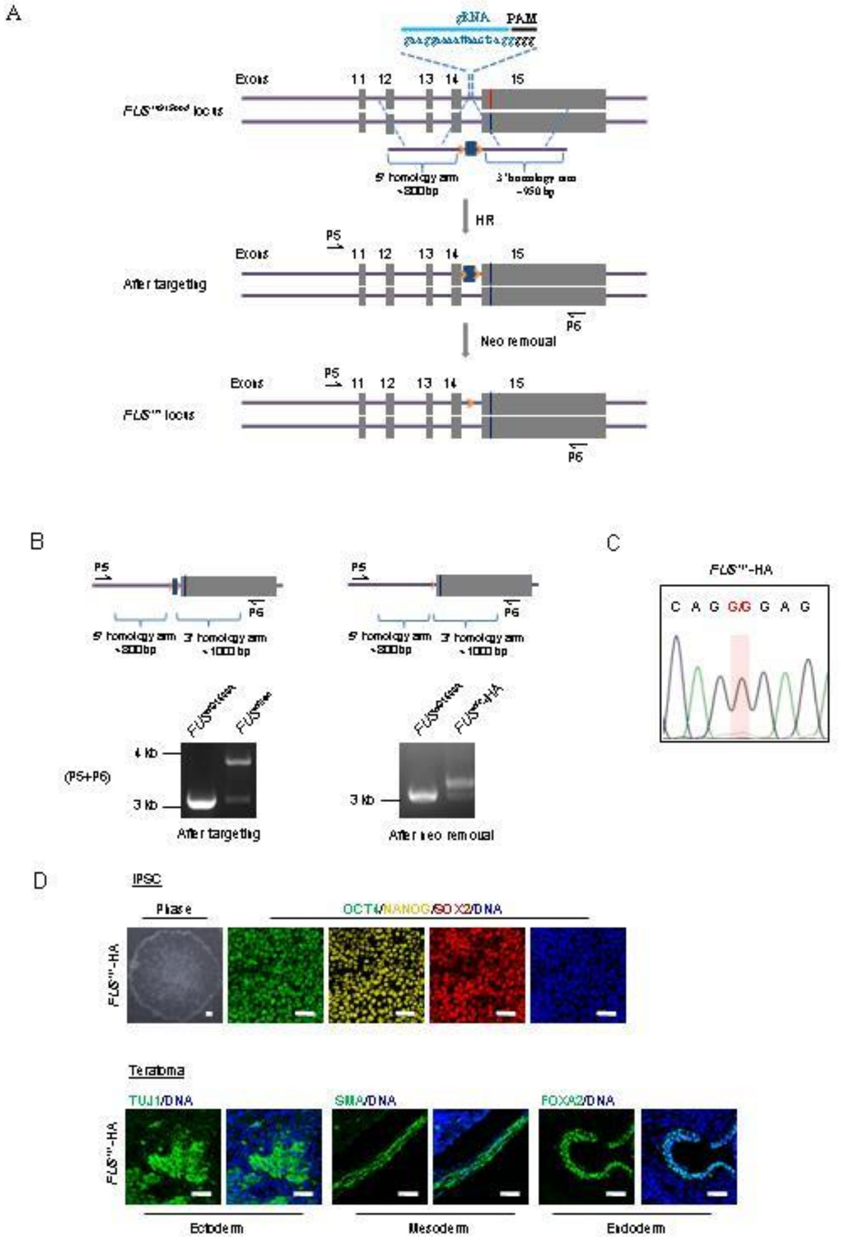

Figure S1. Targeted gene correction of *FUS* mutation using CRISPR/Cas9 system with neo-resisitant homology arms as repair template.

(A) Strategy of correcting *FUS*$^{+/G1566A}$ mutation using neomycin-resistant homology arms as repair template. The sequence of gRNA is shown with the PAM sequence. The blue box is neomycin-resistant cassette. Red line represents the mutant allele, blue line represents the correct allele. Yellow arrow represents the flippase recognition target site. HR, homologous recombination. Primers used for Fig. S1B are shown as arrows (P5, P6).

(B) PCR analysis of targeted clone as indicated (before neo removal (P5+P6): ~ 4 kb. After neo removal (P5+P6): ~ 3 kb). *FUS*$^{+/G1566A}$ iPSC is control. RA: right homology arm. LA: left homology arm. HA: homology arms. *FUS*$^{+/+}$-HA iPSC is corrected clone using neomycin-resistant homology arms as repair template.

(C) DNA sequencing of genomic DNA PCR demonstrates the correction of *FUS*$^{+/G1566A}$ mutation. The red highlight is correct base.

(D) Immunofluorescent images of pluripotent markers, OCT4, NANOG and SOX2 in corrected clone *in vitro* and three-layer markers, TUJ1 (ectoderm), SMA (mesoderm), and FOXA2 (endoderm) in teratomas derived from corrected iPSCs *in vivo*. Nuclei were stained with Hoechst 33342 (blue). Scale bars = 50 μmol/Lm.

Figure S2.

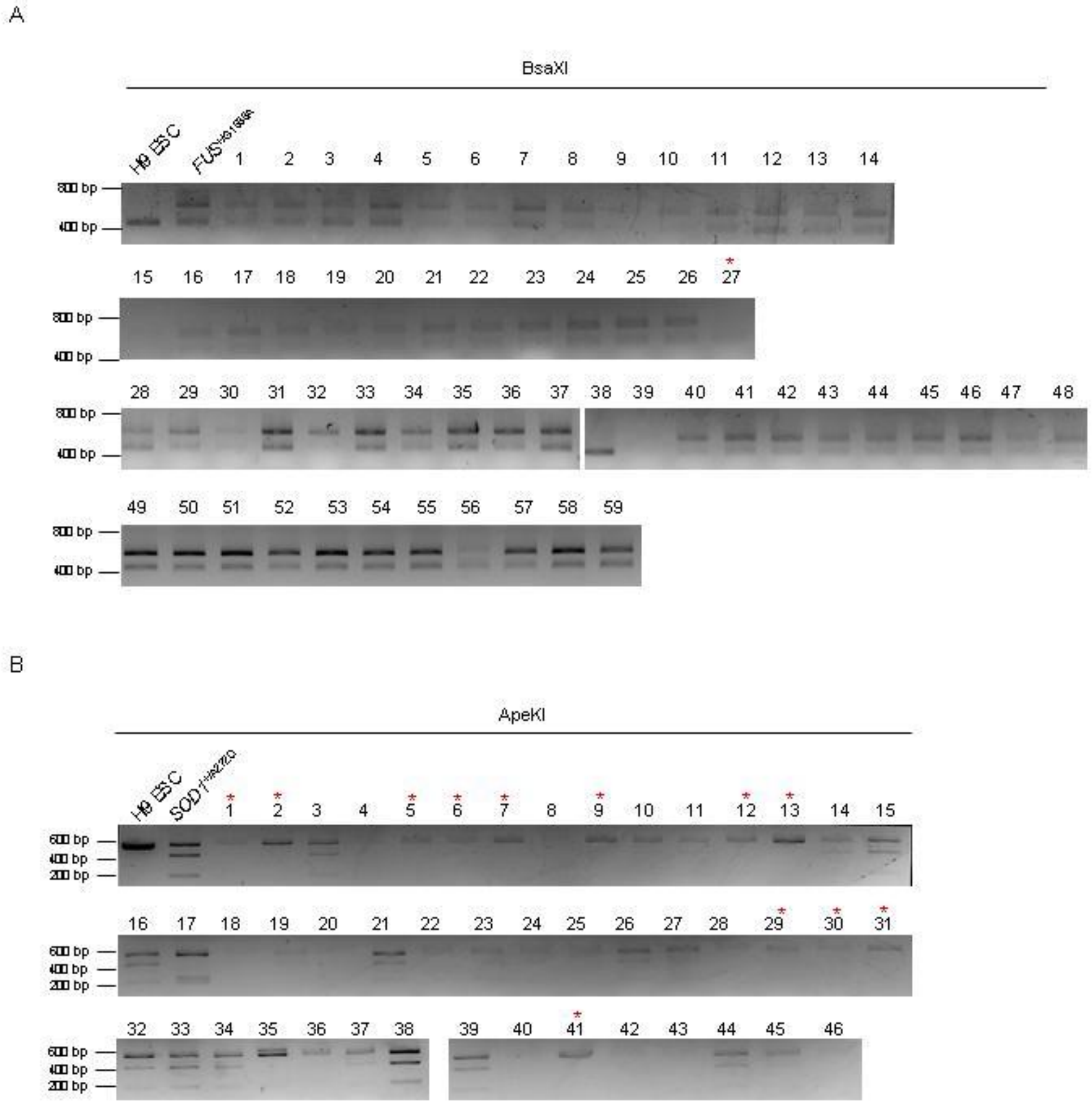

Figure S2. Screening for gene-targeted clones by restriction fragment length polymorphism (RFLP) analysis.

(A) *Bsa*XI restriction digestion of PCR products before and after gene correction. The mutation G>A eliminates *Bsa*XI restriction site that is present in the corrected line. Targeted clones are indicated with red asterisks. Primers used are shown in Fig. 2A**.**

(B) ApeKI restriction digestion of PCR products before and after gene correction. The mutation A>C creates ApeKI restriction site that is absent in the corrected line. Targeted clones are indicated with red asterisks. Primers used are shown in Fig. 2E.

Figure S3.

A

FOT1

TTTTTTAGGACCCAGTTTTGGAGAGCCAGGCTAATTAGTAAGGCTTCTTTATAAAAGTCT TAATCTGTGAAATGTAGCTT

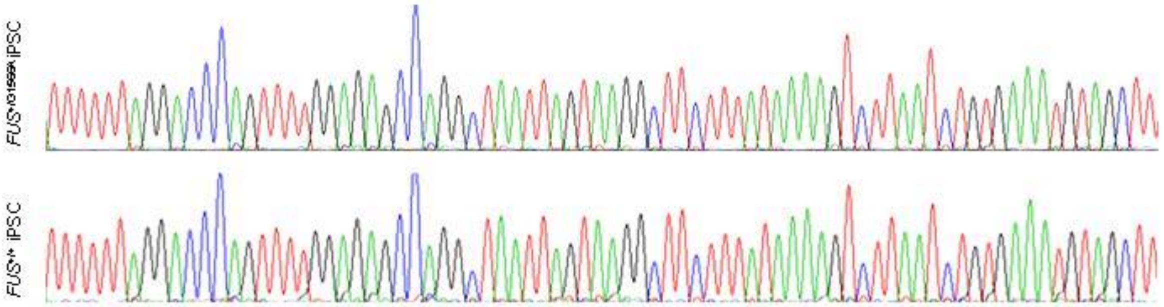

FOT2

TTCTCAATAATGCGTCCATAAGGGGCCAGGTTAATTAATATGGTAACATTGATCTAGGAAAATAATAAATGCAACCAAAC

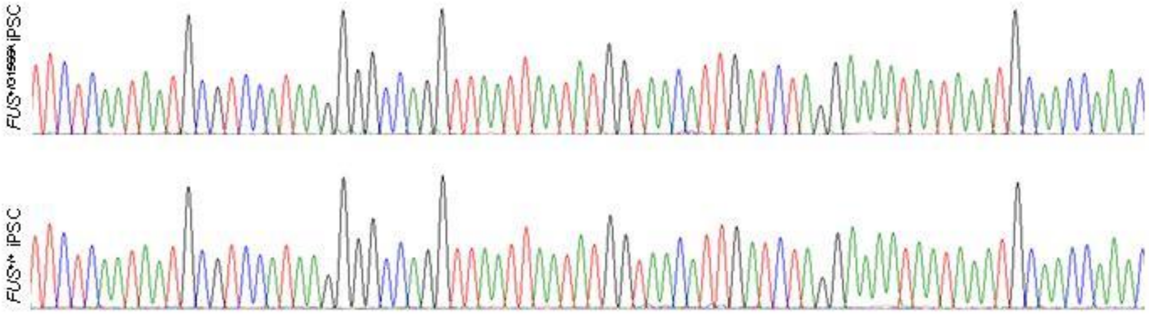

FOT3

ACCAAAATATCCATCCCAAAGAGAAGCTGGCTAATTAATAGAGTGTTCATAGCCTCTAAATTAGATAGGCATAAGATATT

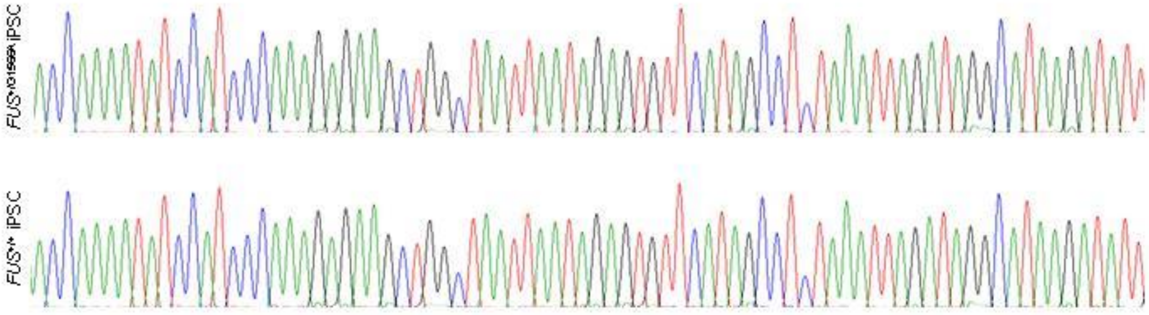

Figure S3.

B

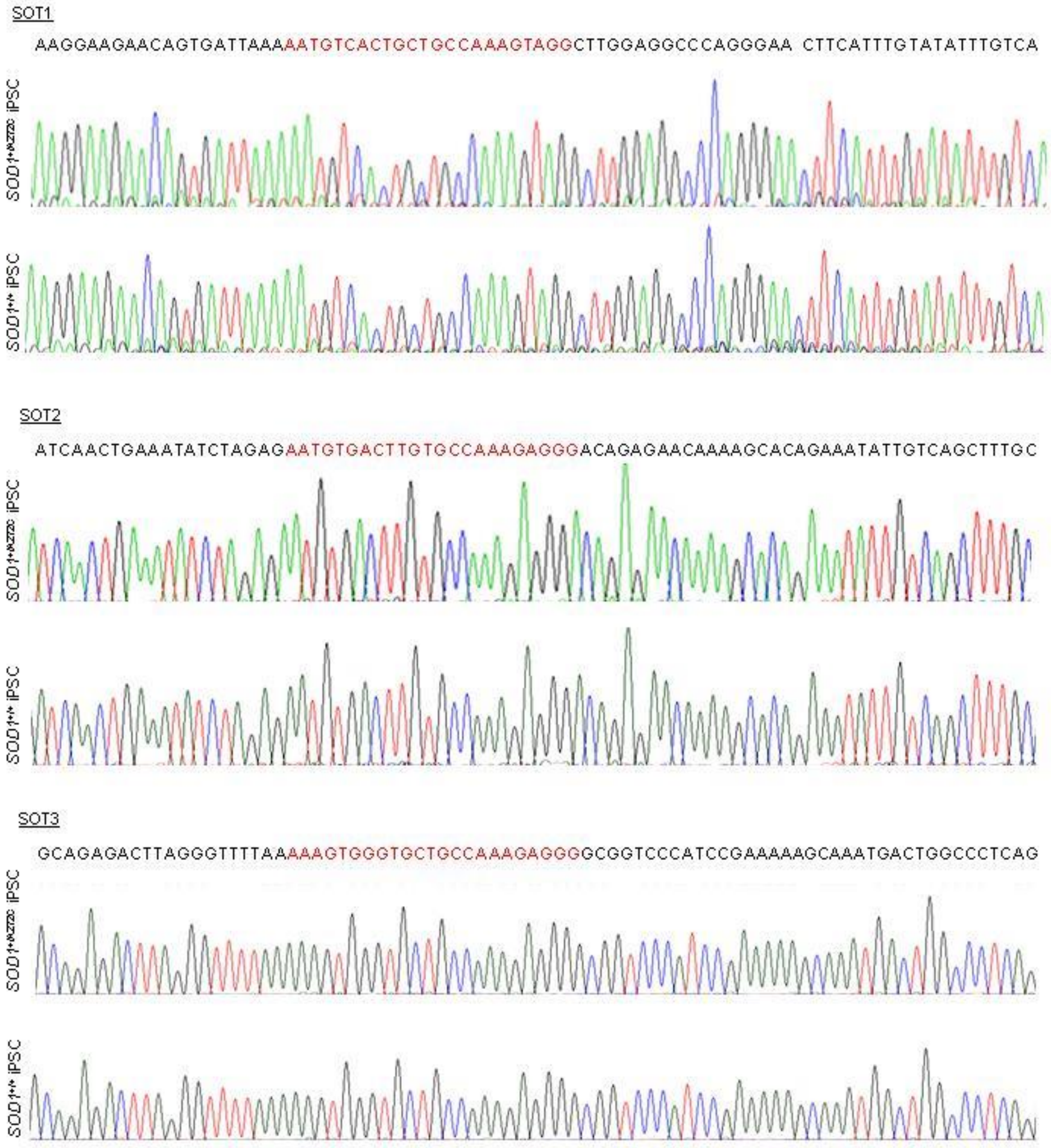

Figure S3. Off-target analysis by DNA sequencing in gene-corrected cells.

(A) DNA sequencing of the top 3 potential off-target sites in *FUS*$^{+/+}$ iPSC. The off-target sites were predicated using an on line tool at http://crispr.genome-engineering.org/. gRNA and PAM sequence are labeled in red. FOT, potential off-target site at gRNA targeted *FUS* gene.

(B) DNA sequencing of the top 3 potential off-target sites in *SOD1*$^{+/+}$ iPSC. The off-target sites were predicated using an on line tool at http://crispr.genome-engineering.org/. gRNA and PAM sequence are labeled in red. SOT, potential off-target site at gRNA targeted *SOD1* gene.

Figure S4.

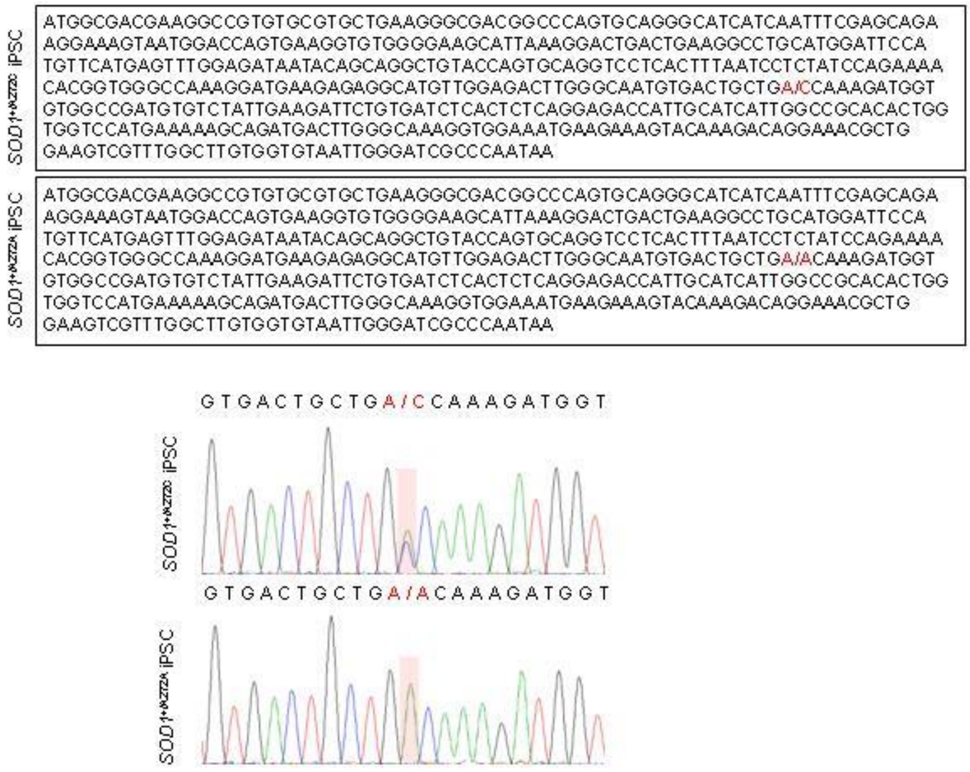

Figure S4. DNA sequencing showing correct gene editing at the coding sequence (CDS) of *SOD1* in ALS-iPSC and corrected iPSC. Mutation (+/A272C) and wildtype (+/A272A) are labeled in red.

Figure S5.

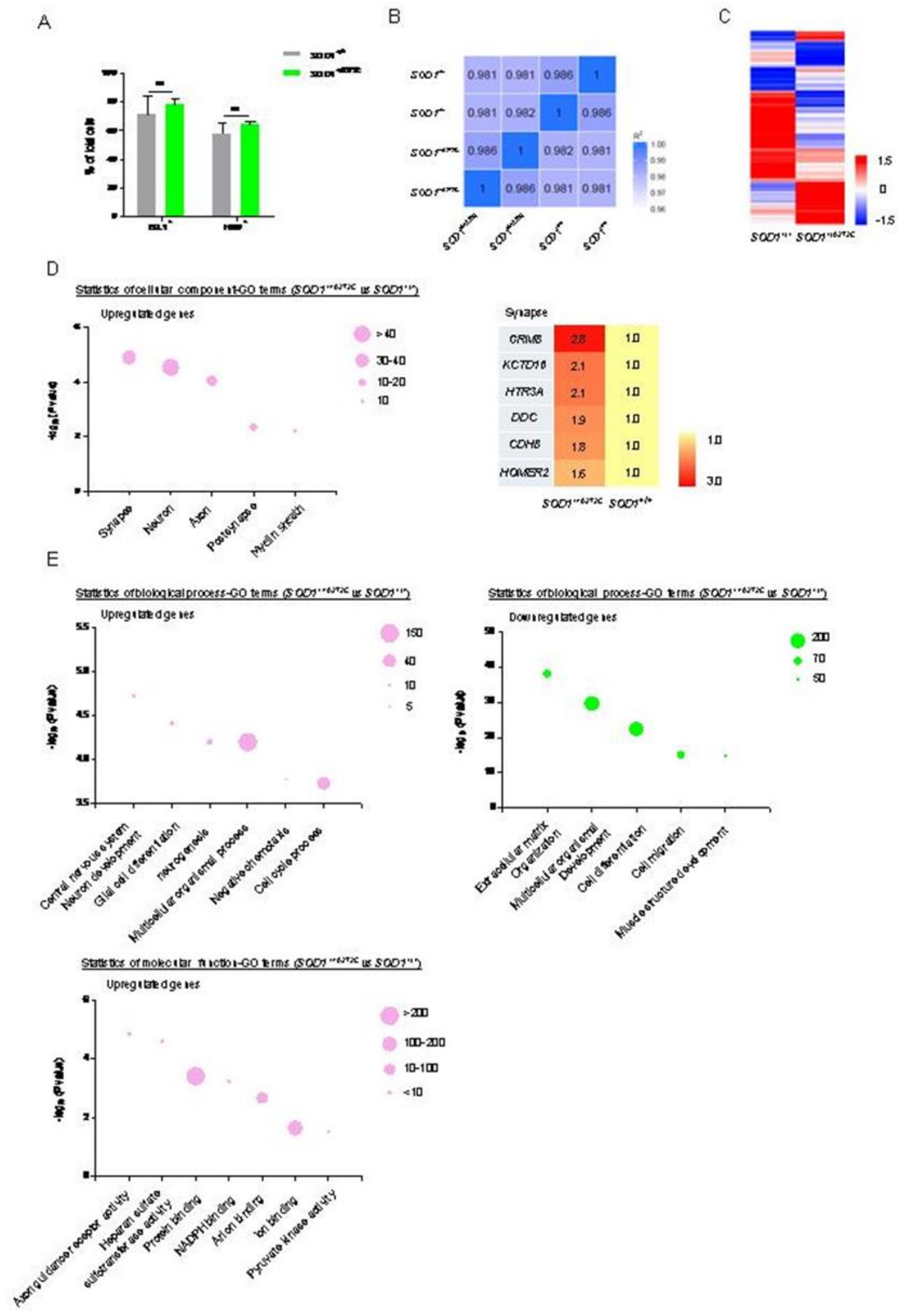

Figure S5. RNA-seq revealed *SOD1*$^{+/A272C}$-affected early pathways underlying ALS.

(A) Percentages of ISL1^{+} or HB9^{+} cells after 12 days differentiation. 300 cells were counted. Data are shown as mean ± SEM. ns, not significant.

(B) The correlation of gene expression between duplicates of *SOD1*$^{+/A272C}$ motor neurons and its isogenic control motor neurons. The Pearson correlation coefficient is shown.

(C) Heat map showing expression levels of differentially expressed genes (q value < 0.05) in *SOD1*$^{+/A272C}$ motor neurons and its isogenic control motor neurons.

(D) GO terms based cellular_component enrichment analysis of the significantly upregulated gene sets in *SOD1*$^{+/A272C}$ motor neurons. Heatmap of upregulated genes between *SOD1*$^{+/A272C}$ motor neurons and its isogenic control motor neurons. Number of altered genes in each GO term is indicated by size of the bubble.

(E) GO terms based biological_process and molecular_function enrichment analysis of the significantly altered gene sets (pink: upregulated genes; green: downregulated genes) in *SOD1*$^{+/A272C}$ motor neurons. Number of altered genes in each GO term is indicated by size of the bubble.

Table S1. The list of mis-regulated genes in *SOD1*$^{+/A272C}$ motor neurons compared with isogenic control.

Table S2. Gene ontology (GO) term analysis of top mis-regulated genes in *SOD1*$^{+/A272C}$ motor neurons compared with isogenic control.

Table S3. Primer list.
